# The clinical, psychosocial, and economic burden of cystic fibrosis lung disease in the era of CFTR modulator therapy

**DOI:** 10.1513/AnnalsATS.202408-800FR

**Published:** 2025-09-17

**Authors:** Isabelle Fajac, Raksha Jain, Marcus A Mall, Bruce K Rubin, Patrick A Flume

**Affiliations:** Université Paris Cité, Faculté de Santé, Paris, France; Assistance Publique–Hôpitaux de Paris, Hôpital Cochin, Service de Physiologie et Explorations Fonctionnelles, Paris, France; Department of Internal Medicine, University of Texas Southwestern Medical Center, Dallas, TX, United States; Department of Pediatric Respiratory Medicine, Immunology and Critical Care Medicine, Charité – Universitätsmedizin Berlin, Berlin, Germany; German Center for Lung Research (DZL), associated partner site Berlin, Berlin, Germany; German Center for Child and Adolescent Health (DZKJ), partner site Berlin, Berlin, Germany; Cluster of Excellence ImmunoPreCept, Charité – Universitätsmedizin Berlin, Berlin, Germany; Department of Pediatrics, Virginia Commonwealth University School of Medicine, Richmond, VA, United States; Departments of Medicine and Pediatrics, Medical University of South Carolina, Charleston, SC, United States

**Keywords:** symptom burden, complications, quality of life, mental health, financial burden

## Abstract

Cystic fibrosis (CF) lung disease imposes a significant clinical, psychosocial, and economic burden on people with CF (pwCF), their caregivers, and healthcare systems. Although the introduction of CF transmembrane conductance regulator (CFTR) modulator therapies has led to significant improvements in symptoms, lung function, exacerbations, and quality of life, substantial burden remains. A subset of pwCF taking CFTR modulator therapy experience residual infection, neutrophilic inflammation (to levels seen in non-CF bronchiectasis), exacerbations, and pulmonary complications. Furthermore, 10%-15% of the global CF population is either ineligible for or intolerant to current CFTR modulator therapies, with some pwCF (although in the minority) experiencing adverse events that necessitate treatment discontinuation. For these people, the burden of disease remains. The worsening of mental health experienced by some pwCF on CFTR modulator therapy adds to the psychosocial burden. Although some evidence suggests a decrease in treatment burden, in general, CFTR modulators have been added to therapeutic regimens rather than replacing symptomatic treatments. While reductions in healthcare resource use have been reported, hospitalizations and emergency department visits, and their associated costs, have not been eliminated. Given the expected improvements in life expectancy following the introduction of these therapies, the burden is likely to continue into old age. A better understanding of the residual clinical, psychosocial, and economic burden that lung disease imposes on pwCF in the era of CFTR modulator therapy highlights the remaining unmet needs and could assist healthcare systems in better planning resource allocation.

## Introduction

Cystic fibrosis (CF) is a genetic disease characterized by cystic fibrosis transmembrane conductance regulator (CFTR) dysfunction.^1-3^ Dysfunction of the CFTR protein, a chloride and bicarbonate channel, leads to the accumulation of hyperconcentrated, viscoelastic, and highly tenacious mucus in the airways. This results in impaired mucociliary clearance, which, in turn, leads to chronic infection and neutrophilic inflammation.^4^ This chronic neutrophilic inflammation invariably leads to progressive structural lung damage, whereby the airways become abnormally dilated, known as bronchiectasis—a typical feature of CF.^3^^,^^5^ As people with CF (pwCF) almost always develop bronchiectasis, we hereafter use the term “CF lung disease” to describe CF-related bronchiectasis, unless otherwise specified.

CFTR modulators bind to the CFTR protein and improve or restore its function.^6^ To date, 5 CFTR modulator therapies have been approved for the treatment of CF, namely ivacaftor, which first became available in 2012^7^; the dual-combination therapies lumacaftor–ivacaftor and tezacaftor–ivacaftor; and the triple-combination therapies elexacaftor–tezacaftor–ivacaftor (ETI) and vanzacaftor–tezacaftor–deutivacaftor (approved by the US Food and Drug Administration [FDA] in December 2024).^8-11^ These therapies have transformed the landscape of clinical care for pwCF through unprecedented improvements in lung function, reduction of pulmonary exacerbation frequency, and improvement in nutritional status, quality of life, and other clinical outcomes, all of which likely translate into increased life expectancy.^12-16^

**Figure 1:**
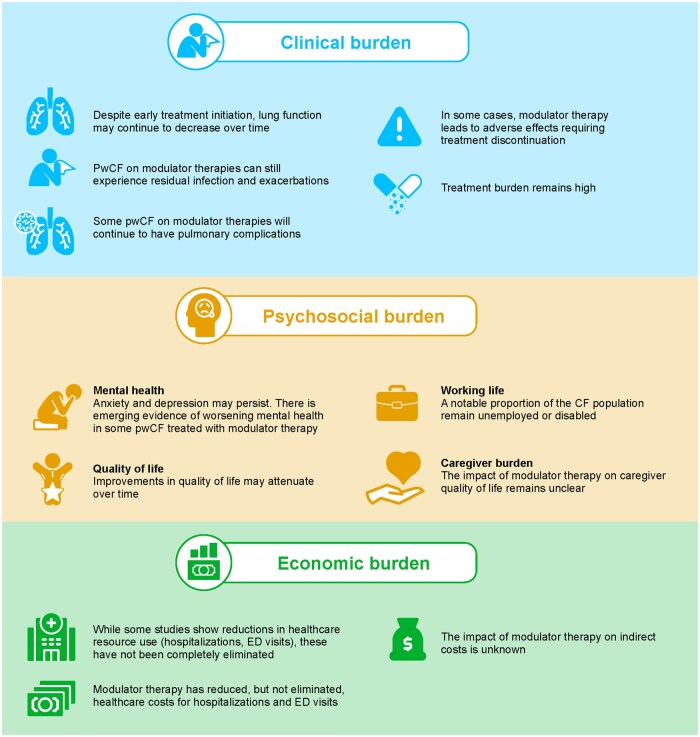
Summary of the clinical, psychosocial, and economic burden of cystic fibrosis lung disease. Abbreviations: CF, cystic fibrosis; ED, emergency department; pwCF, people with cystic fibrosis.

Despite these improvements, data suggest that pwCF taking CFTR modulator therapy are not burden free. This narrative ­review reports on the clinical, psychosocial, and economic burden of CF lung disease in the era of CFTR modulator therapy (Figure 1). A better understanding of this burden could highlight the unmet needs of pwCF lung disease, including the need for new therapies, and assist healthcare systems in planning what resources may be required for pwCF lung disease. This is of particular importance given the aging CF population following the introduction of CFTR modulator therapy.^17^

## Clinical burden of CF lung disease

Particular aspects of the clinical burden of CF lung disease, including symptoms, lung function, infections, pulmonary ­exacerbations, pulmonary complications, treatment burden, and adverse events associated with CFTR modulator therapies, are discussed in the following sections.

### Symptoms and lung function

People with CF experience daily respiratory symptoms including cough, sputum production, dyspnea, and fatigue.^1^^,^^18^ These symptoms exert a physical burden; as such, their reduction is one of the most important outcomes for pwCF.^18^

Treatment with CFTR modulators has been shown to decrease respiratory symptoms and improve lung function in pwCF.^12^ The same is true for people with advanced CF lung disease (generally defined as a forced expiratory volume in 1 second [percent predicted; ppFEV_1_] <40% when stable, referral for lung transplant evaluation, or the presence of other severity markers,^19^ but the definition may differ from study to study), with several studies of ETI showing improvements in ppFEV_1_ and forced vital capacity,^20^^,^^21^ as well as decreased cough and sputum production.^18^^,^^22^ However, there is accumulating evidence to suggest that not all pwCF who are eligible respond to these treatments as expected.^23^ Additionally, more modest improvements in lung function in pwCF with more severe lung disease at baseline are observed compared with the improvements reported in the pivotal clinical trials. This is demonstrated by a study in which the median change in best ppFEV_1_ from 1 year prior to treatment initiation to 1 year after treatment with ETI was 5.1% (compared with up to 13.9% in clinical trials).^24^

Furthermore, lung function may continue to decrease over time despite modulator treatment. For example, in a study assessing the long-term outcomes of ivacaftor treatment in pwCF, although there was an initial improvement in lung function, it continued to decline over time at similar rates to pre-ivacaftor levels, with a decline of 1.82% per annum and a return to baseline lung function by 5 years.^25^ The continued decline in FEV_1_ in adults with CF despite treatment with ivacaftor may be a result of the irreversible lung damage that has already occurred by the time they reach adulthood.^25^ However, this may not be the case for the newer CFTR modulators, with a phase 3 open-label extension study of ETI showing no evidence of pulmonary function loss over a 4-year treatment period,^26^ and a large observational study showing improvements in ppFEV_1_ after 1 and 2 years of treatment with ETI.^27^ Conversely, results from another observational study showed that ppFEV_1_ declined after the initial increase following ETI initiation. This rate of decline was similar to that observed before ETI initiation in adolescents and adults but was significantly worse in younger children (aged 6–11 years).^28^ Given that ETI was approved by the FDA in 2019,^29^ additional data on its long-term effects on lung function are expected to emerge over time.

### Infections

Chronic airway bacterial infection is strongly associated with exacerbations, as well as reduced quality of life and survival in pwCF.^30^*Pseudomonas aeruginosa,* which leads to poorer lung function, higher morbidity, greater risk of hospitalization, and increased mortality,^31-34^ is one of the most dominant pathogens in CF lung disease, including in the era of CFTR modulator therapies. Indeed, *P aeruginosa* infection was reported in 26.0% of pwCF and 59.5% of people with advanced CF lung disease in the United States based on data from the Cystic Fibrosis Foundation [CFF] Patient Registry 2022 Annual Data Report.^35^ In Europe, chronic *P aeruginosa* infection was reported in 30.4% of adults with CF based on data from the European Cystic Fibrosis Society Patient Registry [ECFSPR] 2022 Annual Data Report.^36^ In both registries, most pwCF were receiving CFTR modulators (∼70%),^35^^,^^36^ highlighting the ongoing presence of *P aeruginosa* infection in the era of CFTR modulator therapies.

Several observational studies have shown that 54%-77% of pwCF remain *P aeruginosa* positive on culture following up to 1 year of ETI treatment.^24^ Additionally, a study in pwCF with ­advanced lung disease and chronic *P aeruginosa* infection demonstrated ongoing infection after treatment with ETI or tezacaftor–ivacaftor,^24^ and that the same clonal lineages persisted for up to 21 months following treatment, rather than being eradicated and new infections being established.^24^ A possible explanation for the persistence of *P aeruginosa* is the structural lung damage (CF lung disease/bronchiectasis) that continues to lead to defective pathogen clearance, particularly in the case of pwCF with advanced lung disease.^24^ Cases of *P aeruginosa* acquisition after the initiation of CFTR modulatory therapy have also been reported, suggesting that structural lung damage remains a risk factor for infections.^24^ Limited data are available on the association between changes in *P aeruginosa* status and clinical outcomes after the initiation of ETI treatment.^37^ However, one study found that those who remained *P aeruginosa* positive following ETI initiation had a higher burden of disease in terms of days of intravenous (IV) antibiotic use and days spent hospitalized, even though there was no difference in other clinical outcomes (ppFEV_1_ and nutritional outcomes) between those who remained positive versus those who tested negative following ETI initiation.^37^

Recent observational studies have shown that ETI treatment reduced bacterial density but that chronic infection with *P aeruginosa* or other CF pathogens persists.^38^^,^^39^ Further, this residual infection was found to be associated with residual airway inflammation in adolescents and adults with CF treated with ETI; neutrophilic inflammation was reduced compared with baseline but persisted at levels similar to non-CF bronchiectasis.^39^^,^^40^

Compared with *P aeruginosa*, less is known regarding the impact of CFTR modulators on other infections of importance in pwCF. In one study, CFTR modulator use (ivacaftor, lumacaftor–ivacaftor, or tezacaftor–ivacaftor) was associated with a significant decrease in the risk of nontuberculous mycobacteria (NTM) positivity in pwCF.^41^ Eradication of NTM has also been reported with ETI.^42^ However, the evidence for the effect of CFTR modulators on NTM infections remains limited.^43^ Results from studies examining the effect of ETI on methicillin-sensitive and ­methicillin-resistant *Staphylococcus aureus* detection rates are conflicting.^43^*Burkholderia cepacia* complex (Bcc) is of particular importance in CF due to its negative impact on prognosis, causing chronic progressive lung disease and being a contraindication for lung transplantation at most centers.^44^ However, there are limited data on the effect of CFTR modulators on Bcc. In a small study, tezacaftor–ivacaftor was shown to enhance roscovitine-mediated killing of *Burkholderia cenocepacia*.^45^ In another study, ivacaftor was not associated with a reduced prevalence of Bcc.^46^ Extensive studies investigating the effects of ETI on Bcc have yet to be performed.^44^ As such, further investigations are needed to determine what impact ETI may have on Bcc.^44^

### Pulmonary exacerbations

Lung disease in CF is characterized by pulmonary exacerbations.^47^ These typically manifest as increased cough, sputum production, dyspnea, and lung function decline.^48^ Exacerbations are associated with lung disease progression and a negative impact on quality of life,^33^^,^^49-53^ and are a major cause of morbidity^49^ and increased mortality^54^^,^^55^ in pwCF.

Several clinical trials and real-world studies in pwCF have shown that treatment with CFTR modulator therapies can significantly reduce the risk and frequency of exacerbations.^56-62^ In phase 3 clinical trials, ivacaftor, lumacaftor–ivacaftor, tezacaftor–ivacaftor, and ETI led to relative risk reductions for pulmonary exacerbations of 55%, 39%, 35%, and 63%, respectively, in pwCF.^12^ Real-world studies of ivacaftor, lumacaftor–ivacaftor, and ETI have also shown reductions in the rate of exacerbations in people with advanced CF lung disease.^63-66^

Although pulmonary exacerbations are less frequent in the era of CFTR modulator therapy, they are not eliminated and remain problematic.^67^ Indeed, the reduction in lung function associated with pulmonary exacerbations may not be fully reversed despite treatment.^67^^,^^68^ In one study, ivacaftor did not improve the rate of lung function recovery after pulmonary exacerbations when compared with placebo.^68^ The proportion of pwCF treated with ivacaftor who showed long-term recovery in lung function (46.4%) was similar to that seen in the placebo group (47.7%),^68^ suggesting that treatment with ivacaftor may not have a measurable effect on lung function recovery postexacerbation.^67^^,^^68^ Further research is needed to determine whether this is the case for other CFTR modulators.

### Complications of CF lung disease

Complications of lung disease in CF, including pneumothorax and hemoptysis,^69^ place an additional clinical burden on the people affected. Despite therapeutic advancements including the introduction of CFTR modulator therapies, many pwCF continue to experience complications of pulmonary disease.^69^

Before CFTR modulator therapy, the average annual incidence of pneumothorax was estimated to be 0.64%.^70^ Although less frequent, pwCF continue to experience pneumothorax in the CFTR modulator era, with pneumothorax reported in 0.1% of children and 0.3% of adults in the ECFSPR 2022 Annual Data Report (∼70% of adults and ∼50% of children receiving CFTR modulator therapy)^36^ and in <0.1% of children and 0.1% of adults in the CFF Patient Registry 2022 Annual Data Report (∼70% receiving CFTR modulator therapy).^35^ Pneumothorax contributes significantly to mortality, with 2-year mortality among pwCF with pneumothorax reported to be 48.6%.^69^

Hemoptysis was a common complication before the introduction of CFTR modulator therapy, occurring in approximately 9% of pwCF.^71^ Although less frequent, hemoptysis is still reported in the CFTR modulator era, with 0.3% of children and 2.4% of adults in the CFF Patient Registry 2022 Annual Data Report^35^ reporting hemoptysis. Before CFTR modulator therapy, the average annual incidence of massive hemoptysis, a life-threatening emergency,^72^ was 0.87%.^73^ In the CFTR modulator era, it was reported in <0.1% of children and 0.2% of adults in the CFF Patient Registry 2022 Annual Data Report,^35^ and in 0.2% of children and 1.3% of adults in the ECFSPR 2022 Annual Data Report.^36^ In a recent study in which a large data set from the CFF Patient Registry was assessed, people with any form of hemoptysis were more likely to progress to lung transplant or die without transplant than pwCF who did not have hemoptysis.^74^ In this study, treatment with ivacaftor and lumacaftor–ivacaftor did not appear to change the ­association between hemoptysis and lung transplant or death without transplant; however, tezacaftor–ivacaftor and ETI were not yet approved at the time of the study.^74^ Another study of ivacaftor showed only minimal nominal reductions in pulmonary complications including pneumothorax requiring a chest tube and massive hemoptysis.^75^

Progressive decline in lung function and respiratory failure may require lung transplantation when they become refractory to other therapies.^1^ Treatment with ETI has improved lung function and decreased exacerbations such that some people with advanced CF lung disease that may require lung transplantation are stabilized. In one study, at baseline, all 50 participants met the criteria for either transplant discussion or referral. Following treatment with ETI, improvements in lung function were substantial, and this resulted in recategorizing 7 (14%) people as they no longer met the criteria for transplant discussion or referral.^20^ In another study of 245 people with advanced CF lung disease, ETI treatment was associated with rapid clinical improvement, often leading to the indication for lung transplantation to be suspended.^21^ At treatment initiation, 21.6% of participants were on the lung transplant path; this was reduced to 2% at the end of the follow-up period (median, 84 days).^21^

### Pathology of residual burden of CF lung disease in the era of modulator therapy

CFTR modulator therapies have been shown to ameliorate, but not eliminate, the burden of lung disease in pwCF. This may be because restoration of CFTR function in people with established CF lung disease, although highly effective, is likely limited to anatomically preserved regions of the lung.^15^ Furthermore, CFTR modulators do not fully normalize CFTR function^14^ and lung physiology and do not eliminate chronic neutrophilic inflammation and infection or resolve or reverse chronic lung damage such as bronchiectasis.^1^^,^^30^^,^^76^^,^^77^ Neutrophilic inflammation and infection (key components of the “vicious cycle” of CF lung disease),^78^ as well as the protease-antiprotease imbalance (which plays an important role in the onset and progression of structural lung damage in CF),^39^ may persist following treatment with CFTR modulator therapy. In one study, although restoration of CFTR function by ETI improved chronic airway infection, and reduced neutrophilic inflammation and levels of neutrophil serine proteases (NSPs; neutrophil elastase, proteinase 3, and cathepsin G) over the first year of treatment, this was not to a level similar to that seen in healthy individuals.^39^ Another study found that although treatment with ETI reduced neutrophilic inflammation and NSP levels in the airways of people with established CF lung disease, this was only to levels found in matched non-CF bronchiectasis controls.^40^ Treatment with ETI has also been shown to produce large and rapid reductions in levels of traditional CF pathogens in sputum, but pathogens were not eliminated.^38^ Finally, CFTR modulators do not fully resolve chronic lung damage,^76^^,^^79^ with several studies showing no improvements in wall thickening/bronchiectasis scores, even with reductions in mucus plugging.^77^^,^^80^

### Treatment burden

Reducing treatment burden remains a priority for pwCF, with a 2018 survey identifying this as the top-priority research topic.^81^ Several studies have shown that CFTR modulator therapy can lead to improvements in the acute treatment burden. Both ivacaftor, in pwCF with established lung disease,^25^ and lumacaftor–ivacaftor, in pwCF with severe lung disease,^64^ have decreased the average number of days spent on IV antibiotics following a year of treatment. A decrease in the need for IV antibiotics has also been observed with ETI.^22^ ETI has been shown to significantly reduce the need for maintenance treatments such as long-term oxygen, noninvasive ventilation, and/or enteral tube feeding,^21^ as well as the time spent on airway clearance.^22^ The SIMPLIFY trial showed that discontinuation of daily hypertonic saline or dornase alfa for 6 weeks in pwCF with preserved lung function receiving ETI did not result in clinically meaningful differences in pulmonary function when compared with continuing treatment.^82^ Similarly, the discontinuation of both hypertonic saline and dornase alfa for 6 weeks did not result in any meaningful changes in clinical outcomes compared with those who remained on both therapies.^83^ Data on the effects of discontinuation in people with more severe lung disease are limited, albeit reassuring, with some evidence from a small study supporting the safety of discontinuing hypertonic saline in people with lower lung function (ppFEV_1_ of 40%-59%) being treated with ETI.^84^ Additional insights on the impact of discontinuing chronic daily therapies following initiation of ETI treatment will be provided by studies such as the open-label study CF STORM^85^ and the real-world observational study HERO-2.^86^

Although ETI has perhaps decreased the need for adjunct therapies, a population of pwCF still remains that requires other treatments for residual lung disease. The general consensus is that inhaled antibiotics should not be discontinued in most pwCF on CFTR modulators.^30^ In pwCF with irreversible structural lung damage (which is nearly universally present in older people with CF),^87^ it is expected that adjunct therapies targeting mucociliary clearance, chronic airway infection, and neutrophilic inflammation will still be required.^15^ In general, CFTR modulators, which are generally taken every 12 hours,^8^ have been added to maintenance therapeutic regimens of eligible pwCF, rather than replacing some symptomatic treatments, thus increasing the treatment burden.^88^ Indeed, in a study assessing the treatment burden of CF in the era of CFTR modulator therapy, most respondents (81%) had not stopped another chronic treatment.^89^ Additionally, in a study of posttrial treatment used by pwCF who previously participated in the SIMPLIFY trial discussed above, only 24.6% of people who participated in the hypertonic saline part of the trial and 35.6% of people who participated in the dornase alfa part of the trial reported nonuse of the respective medications at 24 weeks.^90^ In a study assessing the impact of ETI on health-related quality of life in pwCF, Cystic Fibrosis Questionnaire-Revised (CFQ-R) scores for Physical and Respiratory domains were higher than for Emotional and Treatment Burden domains.^91^ Qualitative results indicated that most participants were continuing their pre-ETI treatment regimens or slightly reducing the amount of time spent on their treatments even though they were feeling better, suggesting that treatment burden remains high even with an improvement in physical health.^91^

### Burden of adverse events associated with CFTR modulator therapies

Although in the minority, some pwCF experience significant side effects from CFTR modulators that require them to stop treatment. CFTR modulator therapies are generally well tolerated; however, real-world studies show that the frequency of adverse events and treatment discontinuation may be higher than what has previously been reported in clinical trials. Adverse events such as dyspnea and abnormal respiration have been reported following open-label administration of lumacaftor–ivacaftor in pwCF with severe lung disease, with adverse event rates up to 75%.^92^ Additionally, in up to 35% of pwCF with severe lung disease, ­adverse events were not tolerated and resulted in the discontinuation of treatment.^92^ In a systematic literature review assessing the real-world safety of CFTR modulators, lumacaftor–ivacaftor was associated with a higher frequency of adverse events, particularly respiratory-­related events, compared with the other CFTR modulators.^93^ Dyspnea and chest tightness events with lumacaftor–ivacaftor occurred more frequently in a real-world setting than in clinical trials.^93^ Furthermore, the proportion of pwCF who ­reported respiratory adverse events as the reason for lumacaftor–ivacaftor discontinuation was higher in those with more severe lung disease (74% in pwCF with ppFEV_1_ <40% vs 42% and 29% in pwCF with ppFEV_1_ between 40% and 90% and ≥90%, respectively).^93^ In the same systematic literature review, the most commonly reported side effects observed in studies evaluating ivacaftor included respiratory-related adverse events; elevated transaminases and other hepatic adverse events; gastrointestinal-related adverse events such as abdominal pain, nausea, vomiting, intestinal dysmotility, and gastroenteritis; and headache.^93^ Despite the demonstrated efficacy of ETI in clinical practice, there has been a gradual increase in reports of adverse events.^94^ In a study assessing the ­real-world safety profile of ETI using the FDA’s adverse event ­reporting system, the most common adverse events were ­psychiatric disorders (most commonly anxiety, depression, and insomnia); respiratory, thoracic, and mediastinal disorders (most commonly cough, hemoptysis, rhinorrhea, and increased sputum); gastrointestinal disorders (most commonly abdominal pain and constipation); and infections and infestations.^94^ Headache and increased hepatic enzymes were also commonly reported.^94^ Rash was not an uncommon adverse event reported in real-world studies for ivacaftor, lumacaftor–ivacaftor, and tezacaftor–ivacaftor, with some individuals requiring interruption or discontinuation of treatment for rash or allergic reactions.^93^ An important safety signal identified in the real-world data that was uncommon or not reported in clinical trials was related to mental health.^93^ This is discussed in further detail below.

In summary, pwCF experience residual clinical burden, despite treatment with CFTR modulator therapy. Lung function may continue to decline over time and pwCF on modulator therapy can still experience residual infection and exacerbations. Additionally, some pwCF will continue to have pulmonary complications such as hemoptysis, pneumothorax, and respiratory failure. In some cases, CFTR modulators lead to adverse effects that require treatment discontinuation. In many pwCF, the maintenance treatment burden appears to remain high.

## Psychosocial burden of CF lung disease

Implications outside the clinical sphere are broad ranging, with effects on mental health, quality of life, education and careers, and caregivers.^95^ Importantly, these challenges and burdens may increase with disease progression.^95^

### Mental health

The effect of CFTR modulator therapies on mental health remains poorly understood.^96^ However, high levels of depression and anxiety have been reported in pwCF,^15^ with prevalence estimated to be at least twice that of the general population.^97^ In the CFF Patient Registry Annual Data Report for 2022 (∼70% of people were receiving CFTR modulator therapy), 29.6% and 9.5% of adults (aged ≥18 years) and children (aged <18 years) with CF, respectively, reported depression, while anxiety was reported in 29.4% and 13.7%.^35^ Symptoms of anxiety and depression have been shown to persist in pwCF, despite treatment with CFTR modulator therapy. In one study, treatment with ETI improved symptoms of depression in pwCF; however, anxiety symptoms as measured by the General Anxiety Disorder-7 score did not change compared with baseline after initiation of ETI, and subgroup analysis showed that symptoms of depression were significantly reduced in males but not in females.^98^ In a study conducted by Ramsey and colleagues, pwCF who were taking ETI were found to have depression symptoms that were consistent with the background epidemiology of depression in the CF population,^99^ with the authors suggesting that this refutes any causal association with ETI use. However, these results are questioned in a subsequent editorial, which suggested that a subgroup of pwCF who have worsening depression may have been masked by a group of pwCF who experienced improvement with ETI.^100^ Additionally, the potential confounding effects of the COVID-19 pandemic on the data included in the Ramsey et al. study need to be considered.^100^ Furthermore, emerging evidence has shown worsening of mental health in some pwCF taking CFTR modulator therapy. While this was not reported as an adverse event during phase 3 studies and an extension study, a small number of pwCF taking lumacaftor–ivacaftor have experienced worsening of anxiety and depression, which was, in some cases, associated with suicidal ideation or a suicidal attempt.^96^^,^^101^ There is also some evidence showing that ETI can lead to clinically significant neuropsychiatric adverse events, including worsening of mood, cognition, anxiety, sleep, and suicidal thoughts and behaviors.^102^ This phenomenon is likely multifactorial, with several hypothesized contributors, including direct effects of CFTR modulators on the central nervous system, interactions between CFTR modulators and psychotropic medications, and the psychological impact of starting a new, potentially life-altering medication.^96^ More data on the psychological adverse events associated with CFTR modulator use are likely to emerge with time.

### Impact on quality of life

Health-related quality of life is considerably lower in pwCF than in the general population.^103^ Treatment with ETI can lead to ­improvements in quality of life in people with CF lung disease, with significant increases from baseline in CFQ-R Respiratory Symptoms domain scores after 1 month, 3 months, and 6 months of treatment, and one study showing that the improvement was maintained until approximately 1 year of treatment.^65^^,^^104^^,^^105^ In another study, significant improvements from baseline were observed in CFQ-R Physical Functioning, Body Image, Health Perceptions, Vitality, Respiratory Symptoms, and Treatment Burden domains following 2 years of treatment with ETI.^106^

However, these improvements may not be long lasting, with a study in people with advanced CF lung disease reporting that although improvements in several CFQ-R domain scores were observed in the first year of treatment, these improvements attenuated over time after the first year, with no significant difference from baseline observed after 2 years of treatment.^107^ Additionally, in a study assessing the impact of pulmonary exacerbations on health-related quality of life, in the immediate weeks postexacerbation, improvement was seen in the Emotional Functioning, Respiratory Symptoms, and Health Perceptions domains, whereas further decline was seen for the Eating, Physical Functioning, Role Functioning, Vitality, and Weight domains.^108^ For some measures (eg, Physical Functioning, Vitality), full recovery to preexacerbation levels took several weeks.^108^

### Impact on working life

Advances in clinical care for pwCF have resulted in a substantial increase in life expectancy. As a result, participation in the labor force has increased.^109^ In 2022, approximately three-quarters of adults with CF in the United States reported that they were either studying or had full- or part-time employment as per the CFF Patient Registry 2022 Annual Data Report, in which approximately 70% of people were receiving CFTR modulator therapy.^35^ However, a notable proportion of the CF population remains unemployed or disabled (∼20%).^35^

### Burden on caregivers

Caregiving of pwCF has a wide-ranging impact on caregivers’ quality of life, with scores shown to be lower in caregivers of pwCF compared with the general population.^110^ The burden on caregivers increases with worsening lung disease.^110^ There is limited evidence regarding the effect that CFTR modulators have had on the burden on caregivers of pwCF. While practical improvements have been shown, such as a 5-day decrease in work loss for caregivers of children with CF on lumacaftor–ivacaftor,^111^ improvements in caregiver quality of life have not, as demonstrated in a study assessing health-related quality of life and well-being, in which caregiver-reported health-related quality of life was similar across children with CF who currently, no longer, or never took CFTR modulator therapy.^112^

Overall, there is notable residual psychosocial burden in the era of CFTR modulators. The prevalence of mental health issues such as anxiety and depression are already higher in pwCF than the general population, and this has been shown to persist or worsen in some pwCF taking CFTR modulator therapy. Additionally, while some studies report improvements in quality of life following treatment with CFTR modulators, others show that these ­improvements may not be long lasting. The impact of CFTR modulator therapy on caregiver burden remains unclear.

## Economic burden of CF lung disease

CF lung disease, particularly advanced disease, is associated with increased healthcare resource use,^95^ with most hospitalizations in pwCF being respiratory related.^113^ Some studies have shown a decrease in healthcare resource use with CFTR modulator therapy, with ivacaftor,^25^ lumacaftor–ivacaftor,^58^ and tezacaftor–ivacaftor^114^ reducing the number of days spent in hospital and ETI reducing hospitalizations and emergency department (ED) visits.^115^ However, hospitalizations and ED visits have not been completely eliminated.

CFTR modulator therapy has been shown to reduce, but not eliminate, the cost of hospital and ED visits. For example, in a study that assessed the cost of care in the 180 days before and after ETI initiation, treatment with ETI led to a significant 73% reduction in inpatient hospitalization cost per person (mean ± SD, $16 711 ± $50 590 prior to initiation to $4573 ± $39 470 following initiation) and a significant 41% reduction in total ED visit cost per person ($424 ± $2356 prior to initiation to $248 ± $1441 following initiation of ETI).^115^

Little is known about the impact of CFTR modulator therapy on indirect costs. However, in one study, the point estimates for work absence increased following initiation of treatment with lumacaftor–ivacaftor,^111^ suggesting that the burden still exists post–CFTR modulator therapy.

In summary, CF lung disease places a substantial economic burden on healthcare systems. While some studies show reductions in healthcare resource use and costs (hospitalizations, ED visits), these have not been completely eliminated by CFTR modulator therapies.

## Initiation of CFTR modulator therapy early in life

It is not yet known whether the initiation of CFTR modulator therapy early in life will prevent the development of lung disease.^116^ Studies of ETI in children with CF show a significant rapid and sustained improvement in lung clearance index (LCI),^117^^,^^118^ supporting the potential for greater long-term benefit. However, ETI treatment has been shown to restore CFTR activity in the airways of pwCF to only approximately 40%-50% of normal activity.^14^ A recent study showed that this level of correction of CFTR function does not fully restore airway epithelial homeostasis and host defense in epithelial and immune cells in the airways, not even in children treated as early as 6 years of age.^119^ As such, with the current level of correction (∼40%-50%), pwCF are still expected to develop chronic lung disease (bronchiectasis) even if ETI is started very early. This hypothesis is supported by a natural history study that measured residual CFTR function for a broad spectrum of CFTR genotypes; people with residual CFTR function (∼12%-54% of control epithelia) presented with lung disease, albeit a less severe form than those with absent CFTR function.^120^

New CFTR modulator therapies, to further increase CFTR function above what is currently achieved with ETI, are in ­development.^121^ Indeed, in phase 3 trials, vanzacaftor–tezacaftor–deutivacaftor was shown to outperform ETI in reducing sweat chloride levels (SwCl; a measure of CFTR function).^9^^,^^10^^,^^122^ Pooled data from 2 phase 3 trials revealed an approximately 2 times greater likelihood in the odds of achieving a SwCl level <60 mmol/L (the diagnostic cut-off for a positive test for CF) and an approximately 3 times greater likelihood in the odds of achieving a SwCl level <30 mmol/L (carrier level) for those treated with vanzacaftor–tezacaftor–deutivacaftor compared with ETI.^122^ It is currently unclear what impact these new modulators, such as vanzacaftor–tezacaftor–deutivacaftor (recently approved by the FDA),^11^ may have on reducing the burden of CF lung disease.

Given that CFTR modulators appear generally safe for women and fetuses when taken during pregnancy, in utero treatment via administration to pregnant women is of interest.^123^ Assessment of prenatal treatment with CFTR modulators has focused primarily on gastrointestinal complications of CF; ^123^^,^^124^ as such, the impact on CF lung disease is still to be determined. However, in a case study that monitored the effects of ETI over 1.7 years in an infant exposed to ETI both in utero and postpartum, LCI remained stable in regularly performed measurements every 3-6 months.^125^

## Conclusion

Lung disease places a significant clinical, psychosocial, and economic burden on pwCF and their families, as well as on healthcare systems. Although treatment with CFTR modulator therapies has resulted in a significant reduction in the clinical and psychosocial burden of CF lung disease, a substantial residual burden still exists. Given the expected increases in life expectancy following the introduction of CFTR modulator therapies,^126^ this burden is likely to continue into old age. Additionally, 10%-15% of the global CF population is ineligible for, or intolerant to, current CFTR modulator therapies and the burden of disease remains.^127^ People who are not receiving CFTR modulators, including those who experience adverse effects and cannot tolerate these therapies, may feel like they are being left behind.^128^ Further investigation is needed into the economic implications of CF lung disease in the era of CFTR modulators, alongside its effects on long-term quality of life, mental health, and the well-being of caregivers. New therapies, such as those that target residual neutrophilic inflammation and infection, may complement available treatments for CF such as CFTR modulator therapies, thereby further decreasing the burden of CF lung disease that may now extend into old age.

## Supplementary Material

annalsats.202408-800fr_Supplementary_Data

## Data Availability

This article is open access and distributed under the terms of the Creative Commons Attribution Non-Commercial No Derivatives License 4.0 (http://creativecommons.org/licenses/by-nc-nd/4.0/). For commercial usage and reprints, please contact Diane Gern (dgern@thoracic.org).
