# Supplementary material for: The clinical, psychosocial, and economic burden of cystic fibrosis lung disease in the era of CFTR modulator therapy
Source: Ann Am Thorac Soc. 2025 Sep 17;23(1):17–29. doi: 10.1513/AnnalsATS.202408-800FR (PMC13048512; doi:10.1513/AnnalsATS.202408-800FR)
Supplement: Supplementary file 1 [file annalsats.202408-800fr_supplementary_data.pdf]

ICMJE DISCLOSURE FORM

Date: 26 July 2024
Your Name: Isabelle Fajac
Project: The clinical, emotional and economic burden of cystic fibrosis lung disease

In the interest of transparency, we ask you to disclose all relationships/activities/interests listed below that are related to the content of your manuscript. "Related" means any relation with for-profit or not-for-profit third parties whose interests may be affected by the content of the manuscript. Disclosure represents a commitment to transparency and does not necessarily indicate a bias. If you are in doubt about whether to list a relationship/activity/interest, it is preferable that you do so.

The following questions apply to the author's relationships/activities/interests as they relate to the current manuscript only.

The author's relationships/activities/interests should be defined broadly. For example, if your manuscript pertains to the epidemiology of hypertension, you should declare all relationships with manufacturers of antihypertensive medication, even if that medication is not mentioned in the manuscript.

In item #1 below, report all support for the work reported in this manuscript without time limit. For all other items, the time frame for disclosure is the past 36 months.

		**Name all entities with whom you have this relationship or indicate none (add rows as needed)**	**Specifications/Comments (e.g., if payments were made to you or to your institution)**
**Time frame: Since the initial planning of the work**			
1	All support for the present manuscript (e.g., funding, provision of study materials, medical writing, article processing charges, etc.) **No time limit for this item.**	____ None	
		Nucleus Global	Writing/submission support, funded by Boehringer Ingelheim
**Time frame: past 36 months**			
2	Grants or contracts from any entity (if not indicated in item #1 above).	__X__ None	
3	Royalties or licenses	__X__ None	
4	Consulting fees	__ __ None	

		AbbVie	Personal
		Boehringer Ingelheim	Personal
		Genvade	Personal
		Kither Biotech	Personal
		Vertex Pharmaceuticals	Personal
5	Payment or honoraria for lectures, presentations, speakers bureaus, manuscript writing or educational events	__X___ None	
6	Payment for expert testimony	__X__ None	
7	Support for attending meetings and/or travel	__X__ None	
8	Patents planned, issued or pending	__X__ None	
9	Participation on a Data Safety Monitoring Board or Advisory Board	__X__ None	
10	Leadership or fiduciary role in other board, society, committee or advocacy group, paid or unpaid	__X__ None	
11	Stock or stock options	__X__ None	
12	Receipt of equipment, materials, drugs, medical writing, gifts or other services	__X__ None	
13	Other financial or non-financial interests	__X__ None	

Please place an "X" next to the following statement to indicate your agreement:

___ I certify that I have answered every question and have not altered the wording of any of the questions on this form.

ICMJE DISCLOSURE FORM

Date: 26 July 2024
Your Name: Patrick A Flume
Project: The clinical, emotional and economic burden of cystic fibrosis lung disease

In the interest of transparency, we ask you to disclose all relationships/activities/interests listed below that are related to the content of your manuscript. "Related" means any relation with for-profit or not-for-profit third parties whose interests may be affected by the content of the manuscript. Disclosure represents a commitment to transparency and does not necessarily indicate a bias. If you are in doubt about whether to list a relationship/activity/interest, it is preferable that you do so.

The following questions apply to the author's relationships/activities/interests as they relate to the current manuscript only.

The author's relationships/activities/interests should be defined broadly. For example, if your manuscript pertains to the epidemiology of hypertension, you should declare all relationships with manufacturers of antihypertensive medication, even if that medication is not mentioned in the manuscript.

In item #1 below, report all support for the work reported in this manuscript without time limit. For all other items, the time frame for disclosure is the past 36 months.

		**Name all entities with whom you have this relationship or indicate none (add rows as needed)**	**Specifications/Comments (e.g., if payments were made to you or to your institution)**
		**Time frame: Since the initial planning of the work**	
1	All support for the present manuscript (e.g., funding, provision of study materials, medical writing, article processing charges, etc.) **No time limit for this item.**	____ None	
		Boehringer Ingelheim	Study sponsor
		Nucleus Global	Medical writing assistance, in the form of the preparation and revision of the manuscript, supported financially by Boehringer Ingelheim
		**Time frame: past 36 months**	
2	Grants or contracts from any entity (if not indicated in item #1 above).	Armata	
		Boehringer Ingelheim	
		Cystic Fibrosis Foundation Therapeutics	
		National Institutes of Health	
		Sound Pharmaceuticals	
		Vertex Pharmaceuticals	
3	Royalties or licenses	____ None	

4	Consulting fees	Eloxx Pharmaceuticals	
		Ionis Pharmaceuticals	
		Sionna	
		Vertex Pharmaceuticals	
5	Payment or honoraria for lectures, presentations, speakers bureaus, manuscript writing or educational events	____ None	
6	Payment for expert testimony	____ None	
7	Support for attending meetings and/or travel	____ None	
8	Patents planned, issued or pending	____ None	
9	Participation on a Data Safety Monitoring Board or Advisory Board	____ None	
10	Leadership or fiduciary role in other board, society, committee or advocacy group, paid or unpaid	____ None	
11	Stock or stock options	____ None	
12	Receipt of equipment, materials, drugs, medical writing, gifts or other services	____ None	
13	Other financial or non-financial interests	____ None	

Please place an "X" next to the following statement to indicate your agreement:

__X__ I certify that I have answered every question and have not altered the wording of any of the questions on this form.

ICMJE DISCLOSURE FORM

Date: 26 July 2024
Your Name: Raksha Jain
Project: The clinical, emotional and economic burden of cystic fibrosis lung disease

In the interest of transparency, we ask you to disclose all relationships/activities/interests listed below that are related to the content of your manuscript. "Related" means any relation with for-profit or not-for-profit third parties whose interests may be affected by the content of the manuscript. Disclosure represents a commitment to transparency and does not necessarily indicate a bias. If you are in doubt about whether to list a relationship/activity/interest, it is preferable that you do so.

The following questions apply to the author's relationships/activities/interests as they relate to the current manuscript only.

The author's relationships/activities/interests should be defined broadly. For example, if your manuscript pertains to the epidemiology of hypertension, you should declare all relationships with manufacturers of antihypertensive medication, even if that medication is not mentioned in the manuscript.

In item #1 below, report all support for the work reported in this manuscript without time limit. For all other items, the time frame for disclosure is the past 36 months.

		**Name all entities with whom you have this relationship or indicate none (add rows as needed)**	**Specifications/Comments (e.g., if payments were made to you or to your institution)**
		**Time frame: Since the initial planning of the work**	
1	All support for the present manuscript (e.g., funding, provision of study materials, medical writing, article processing charges, etc.) **No time limit for this item.**	____ None	
		Nucleus Global	Writing/submission support, funded by Boehringer Ingelheim
		**Time frame: past 36 months**	
2	Grants or contracts from any entity (if not indicated in item #1 above).	__X__ None	
3	Royalties or licenses	__X__ None	
4	Consulting fees	____ None	Boehringer Ingelheim CF and bronchiectasis clinical trial design steering commitee

5	Payment or honoraria for lectures, presentations, speakers bureaus, manuscript writing or educational events	__X___ None	
6	Payment for expert testimony	__X__ None	
7	Support for attending meetings and/or travel	__X__ None	
8	Patents planned, issued or pending	__X__ None	
9	Participation on a Data Safety Monitoring Board or Advisory Board	__X__ None	
10	Leadership or fiduciary role in other board, society, committee or advocacy group, paid or unpaid	__X__ None	
11	Stock or stock options	__X__ None	
12	Receipt of equipment, materials, drugs, medical writing, gifts or other services	__X__ None	
13	Other financial or non-financial interests	__X__ None	

Please place an "X" next to the following statement to indicate your agreement:

___ I certify that I have answered every question and have not altered the wording of any of the questions on this form.

ICMJE DISCLOSURE FORM

Date: 26 July 2024

Your Name: Marcus A Mall

Manuscript Title: **The clinical, emotional and economic burden of cystic fibrosis lung disease**

Manuscript Number (if known): N/A

In the interest of transparency, we ask you to disclose all relationships/activities/interests listed below that are related to the content of your manuscript. "Related" means any relation with for-profit or not-for-profit third parties whose interests may be affected by the content of the manuscript. Disclosure represents a commitment to transparency and does not necessarily indicate a bias. If you are in doubt about whether to list a relationship/activity/interest, it is preferable that you do so.

The author's relationships/activities/interests should be defined broadly. For example, if your manuscript pertains to the epidemiology of hypertension, you should declare all relationships with manufacturers of antihypertensive medication, even if that medication is not mentioned in the manuscript.

In item #1 below, report all support for the work reported in this manuscript without time limit. For all other items, the time frame for disclosure is the past 36 months.

		Name all entities with whom you have this relationship or indicate none (add rows as needed)	Specifications/Comments (e.g., if payments were made to you or to your institution)
Time frame: Since the initial planning of the work			
1	All support for the present manuscript (e.g., funding, provision of study materials, medical writing, article processing charges, etc.) **No time limit for this item.**	☐ None	
		Boehringer Ingelheim	Medical writing and editorial support, no payments made
			Click the tab key to add additional rows.
Time frame: past 36 months			
**2**	Grants or contracts from any entity (if not indicated in item #1 above).	☐ **None**	
		German Research Foundation (DFG)	SFB-TR 84, SFB-1449 project #431232613, and project #450557679, payments to institution
		German Ministry for Education and Research (BMBF)	Grant #82DZL009B1, #82DZL009C1 and 01GL2401A, payments to institution
		German Innovation Fund	01NVF19008, payments to institution
		Vertex Pharmaceuticals	Independent medical grant and contracts for participation in clinical trials, payments to institution
		Boehringer Ingelheim	Contracts for participation in clinical trials, payments to institution

		Name all entities with whom you have this relationship or indicate none (add rows as needed)	Specifications/Comments (e.g., if payments were made to you or to your institution)
**3**	Royalties or licenses	☒ **None**	
**4**	Consulting fees	☐ **None**	
		Abbvie	Personal fees for consultancy
		Boehringer Ingelheim	Personal fees for consultancy
		Enterprise Therapeutics	Personal fees for consultancy
		Kither Biotec	Personal fees for consultancy
		Prieris	Personal fees for consultancy
		Recode	Personal fees for consultancy
		Splisense	Personal fees for consultancy
		Vertex Pharmaceuticals	Personal fees for consultancy
**5**	Payment or honoraria for lectures, presentations, speakers bureaus, manuscript writing or educational events	☐ **None**	
		Vertex Pharmaceuticals	Honoraria for presentations and educational events
**6**	Payment for expert testimony	☒ **None**	
**7**	Support for attending meetings and/or travel	☐ **None**	
		Boehringer Ingelheim	Travel reimbursement for participation in advisory board meetings
		Vertex Pharmaceuticals	Travel reimbursement for participation in advisory board meetings
**8**	Patents planned, issued or pending	☒ **None**	
**9**	Participation on a Data Safety Monitoring	☐ **None**	
		Abbvie	Personal fees for participation in advisory board
		Boehringer Ingelheim	Personal fees for participation in advisory board

		Name all entities with whom you have this relationship or indicate none (add rows as needed)	Specifications/Comments (e.g., if payments were made to you or to your institution)
	Board or Advisory Board	Enterprise Therapeutics	Personal fees for participation in advisory board
		Kither Biotec	Personal fees for participation in advisory board
		Pari	Personal fees for participation in advisory board
		Vertex Pharmaceuticals	Personal fees for participation in advisory board
**10**	Leadership or fiduciary role in other board, society, committee or advocacy group, paid or unpaid	☐ **None**	
		Fellow of ERS (FERS)	Unpaid
**11**	Stock or stock options	☒ **None**	
**12**	Receipt of equipment, materials, drugs, medical writing, gifts or other services	☒ **None**	
**13**	Other financial or non-financial interests	☒ **None**	

Please place an "X" next to the following statement to indicate your agreement:

☐ I certify that I have answered every question and have not altered the wording of any of the questions on this form.

ICMJE DISCLOSURE FORM

Date: 26 July 2024
Your Name: Bruce K Rubin
Project: The clinical, emotional and economic burden of cystic fibrosis lung disease

In the interest of transparency, we ask you to disclose all relationships/activities/interests listed below that are related to the content of your manuscript. "Related" means any relation with for-profit or not-for-profit third parties whose interests may be affected by the content of the manuscript. Disclosure represents a commitment to transparency and does not necessarily indicate a bias. If you are in doubt about whether to list a relationship/activity/interest, it is preferable that you do so.

The following questions apply to the author's relationships/activities/interests as they relate to the current manuscript only.

The author's relationships/activities/interests should be defined broadly. For example, if your manuscript pertains to the epidemiology of hypertension, you should declare all relationships with manufacturers of antihypertensive medication, even if that medication is not mentioned in the manuscript.

In item #1 below, report all support for the work reported in this manuscript without time limit. For all other items, the time frame for disclosure is the past 36 months.

		**Name all entities with whom you have this relationship or indicate none (add rows as needed)**	**Specifications/Comments (e.g., if payments were made to you or to your institution)**
**Time frame: Since the initial planning of the work**			
1	All support for the present manuscript (e.g., funding, provision of study materials, medical writing, article processing charges, etc.) **No time limit for this item.**	____ None	
		Nucleus Global	Writing/submission support, funded by Boehringer Ingelheim
**Time frame: past 36 months**			
2	Grants or contracts from any entity (if not indicated in item #1 above).	____ None	
3	Royalties or licenses	____ None	
4	Consulting fees	____ Boehringer Ingelheim	Payments made to me

5	Payment or honoraria for lectures, presentations, speakers bureaus, manuscript writing or educational events	______ None	
6	Payment for expert testimony	____None	
7	Support for attending meetings and/or travel	_____ None	
8	Patents planned, issued or pending	_____ None	
9	Participation on a Data Safety Monitoring Board or Advisory Board	_____ None	
10	Leadership or fiduciary role in other board, society, committee or advocacy group, paid or unpaid	_____ None	
11	Stock or stock options	_____ None	
12	Receipt of equipment, materials, drugs, medical writing, gifts or other services	_____ None	
13	Other financial or non-financial interests	_____ None	

Please place an "X" next to the following statement to indicate your agreement:

_x__ I certify that I have answered every question and have not altered the wording of any of the questions on this form.
